# Whole Genome Analysis of *Leptospira licerasiae* Provides Insight into Leptospiral Evolution and Pathogenicity

**DOI:** 10.1371/journal.pntd.0001853

**Published:** 2012-10-25

**Authors:** Jessica N. Ricaldi, Derrick E. Fouts, Jeremy D. Selengut, Derek M. Harkins, Kailash P. Patra, Angelo Moreno, Jason S. Lehmann, Janaki Purushe, Ravi Sanka, Michael Torres, Nicholas J. Webster, Joseph M. Vinetz, Michael A. Matthias

**Affiliations:** 1 Instituto de Medicina Tropical Alexander von Humboldt, Universidad Peruana Cayetano Heredia, Lima, Peru; 2 Division of Infectious Diseases, Department of Medicine, University of California San Diego School of Medicine, La Jolla, California, United States of America; 3 J. Craig Venter Institute, Rockville, Maryland, United States of America; 4 Departamento de Ciencias Celulares y Moleculares, Laboratorio de Investigación y Desarrollo, Facultad de Ciencias, Universidad Peruana Cayetano Heredia, Lima, Peru; 5 Department of Medicine, University of California San Diego School of Medicine, La Jolla, California, United States of America; Institut Pasteur, France

## Abstract

The whole genome analysis of two strains of the first intermediately pathogenic leptospiral species to be sequenced (*Leptospira licerasiae* strains VAR010 and MMD0835) provides insight into their pathogenic potential and deepens our understanding of leptospiral evolution. Comparative analysis of eight leptospiral genomes shows the existence of a core leptospiral genome comprising 1547 genes and 452 conserved genes restricted to infectious species (including *L. licerasiae*) that are likely to be pathogenicity-related. Comparisons of the functional content of the genomes suggests that *L. licerasiae* retains several proteins related to nitrogen, amino acid and carbohydrate metabolism which might help to explain why these *Leptospira* grow well in artificial media compared with pathogenic species. *L. licerasiae* strains VAR010^T^ and MMD0835 possess two prophage elements. While one element is circular and shares homology with LE1 of *L. biflexa*, the second is cryptic and homologous to a previously identified but unnamed region in *L. interrogans* serovars Copenhageni and Lai. We also report a unique O-antigen locus in *L. licerasiae* comprised of a 6-gene cluster that is unexpectedly short compared with *L. interrogans* in which analogous regions may include >90 such genes. Sequence homology searches suggest that these genes were acquired by lateral gene transfer (LGT). Furthermore, seven putative genomic islands ranging in size from 5 to 36 kb are present also suggestive of antecedent LGT. How *Leptospira* become naturally competent remains to be determined, but considering the phylogenetic origins of the genes comprising the O-antigen cluster and other putative laterally transferred genes, *L. licerasiae* must be able to exchange genetic material with non-invasive environmental bacteria. The data presented here demonstrate that *L. licerasiae* is genetically more closely related to pathogenic than to saprophytic *Leptospira* and provide insight into the genomic bases for its infectiousness and its unique antigenic characteristics.

## Author Summary

Leptospirosis is one of the most common diseases transmitted by animals worldwide and is important because it is a major cause of febrile illness in tropical areas and also occurs in epidemic form associated with natural disasters and flooding. The mechanisms through which *Leptospira* cause disease are not well understood. In this study we have sequenced the genomes of two strains of *Leptospira licerasiae* isolated from a person and a marsupial in the Peruvian Amazon. These strains were thought to be able to cause only mild disease in humans. We have compared these genomes with other leptospires that can cause severe illness and death and another leptospire that does not infect humans or animals. These comparisons have allowed us to demonstrate similarities among the disease-causing *Leptospira*. Studying genes that are common among infectious strains will allow us to identify genetic factors necessary for infecting, causing disease and determining the severity of disease. We have also found that *L. licerasiae* seems to be able to uptake and incorporate genetic information from other bacteria found in the environment. This information will allow us to begin to understand how *Leptospira* species have evolved.

## Introduction

Leptospirosis is a globally important tropical infectious disease that takes a disproportionate toll in tropical regions [1]. Caused by more than 250 serovars of spirochetes distributed among nine species of pathogenic *Leptospira* and at least five known species of intermediate *Leptospira* [2], the burden of leptospirosis disease falls predominantly on people living in poverty and under inadequate sanitary conditions [3]. Yet, pathogenic mechanisms in leptospirosis remain poorly understood [2]. Reasons for the varying pathogenic potentials of different varieties of *Leptospira* to cause human disease have not been explored. Mechanisms of leptospiral tropisms for different mammalian reservoirs hosts are unknown. Lateral transfer of DNA has been observed in *Leptospira* but mechanisms for such transfer have yet to be defined [4]–[6]. The present study was designed to gain insight into the evolution of intermediate *Leptospira* with the highest degree of resolution currently possible—using comparative whole genome analysis—and to explore the degree to which evidence might link this leptospiral clade to an evolutionary position between pathogenic and saprophytic *Leptospira* clades as suggested by phylogenetic analysis of 16S rRNA gene sequences [7]–[9].

DNA-relatedness and phylogenetic analyses have resolved the genus *Leptospira* into three distinct lineages [8], [10]–[14] comprising 20 species: nine pathogens, five intermediates and six saprophytes. Pathogenic *Leptospira* are capable of infecting and causing disease in humans and animals; intermediate *Leptospira* are able to infect humans and animals and cause a variety of clinical manifestations [8], [15], [16], although less frequently; saprophytic *Leptospira* are environmental bacteria that do not infect mammals at all. Genome sequencing efforts have so far focused on pathogenic (*L. interrogans* [17], [18] and *L. borgpetersenii* [19]) and saprophytic species (*L. biflexa* [20]). Genomic comparisons indicate that while the *L. biflexa* genome is relatively stable, the genomes of pathogenic species have undergone considerable insertion sequence-mediated rearrangement [19], [20]. It has been shown that there is considerable genomic plasticity even within the same species. For example, an ∼54 kb genomic island and a large inversion in Chromosome I differentiate the *L. interrogans* sv. Lai and Copenhageni genomes [18], whose coding sequences are ∼99% similar at the amino acid level. A comparison of *in vitro* growth characteristics also indicates that the third lineage of *Leptospira*, which includes *L. licerasiae*, occupies an intermediate position between the pathogenic and saprophytic species. Despite reference to intermediate *Leptospira* as “saprophytic intermediates,” [9] convincing clinical data confirm the pathogenicity of these *Leptospira* [8], [13]. Knowledge of the genomic content of these intermediate species is necessary to complete our understanding of leptospiral evolution.

In this study, we sequenced and annotated genomes of *L. licerasiae* sv. Varillal strains VAR010 and MMD0835, the first intermediate species to be sequenced. In view of the range of stresses encountered by pathogenic bacteria during the course of infection, it is becoming apparent that in addition to virulence factors such as hemolysins there are additional proteins or contributory (pathogenicity-associated) factors involved in stress management strategies that are essential for successful infection. Genomic comparisons of the infectious species *L. licerasiae*, *L. interrogans*, *L. borgpetersenii* and the non-infectious saprophyte *L. biflexa* have provided much needed insight into these contributory factors, leptospiral virulence and pathogenicity.

## Methods

### Bacterial strain and genomic DNA extraction

*L. licerasiae* sv. Varillal type strain VAR010^T^ (human isolate) and strain MMD0835 (*Philander* isolate) were originally isolated in Iquitos, Peru [8]. The type strain has been deposited in the American Type Culture Collection (ATCC BAA-1110^T^). *L. licerasiae* sv. Varillal str. MMD0835 strain is available through BEI Resources (http://www.beiresources.org/). Both strains were grown in liquid Ellinghausen-McCullough-Johnson-Harris (EMJH) medium under standard culture conditions to a density of ∼10^8^ organisms/mL. Cells were harvested from 10 mL of culture (10^9^ *Leptospira*) by centrifugation and genomic DNA (gDNA) was extracted using TRIzol (Invitrogen Life Technologies, USA) following manufacturer's directions. To remove RNA, extracted gDNA was then treated with an RNase cocktail (Roche, USA) containing RNase A and H.

### Genome sequencing and assembly

The genome of *L. licerasiae* sv. Varillal type strain VAR010^T^ was sequenced using a combination of 454 FLX Titanium and Illumina Solexa Genome Analyzer IIX. Paired-end libraries were constructed with fragment sizes ranging from 2000 to 4000 for 454 and 200 to 300 for Illumina. A total of 2272294 reads (1:4.26 454:Illumina) were assembled using the Celera Assembler version 7.0beta [21]. The genome assembled into 14 contigs (4 scaffolds) at 58-fold sequence coverage with 99.93% of the genome with more than 19-fold coverage. *L. licerasiae* sv. Varillal str. MMD0835 was sequenced using just the Illumina Genome Analyzer II platform. A single paired-end library with a fragment size between 300–500 bp was constructed. A total of 1112438 reads were used by the CLC bio *de novo* assembler (CLC NGS Cell v. 3.20.50819, http://www.clcbio.com) to generate 48 contigs at 25-fold sequence coverage with 78.0% of the genome above 19-fold coverage (99.9% above 4-fold coverage).

### Deposition of Genome Sequence Data

The nucleotide sequences and the corresponding automated annotations for the genomes of *L. licerasiae* str. VAR010^T^ and MMD0835 were submitted to GenBank, with accession numbers AHOO01000000 and NZ_AFLO00000000, respectively.

### Annotation

Genomes were run through the JCVI automated annotation pipeline v10.0. *Ab initio* gene predictions were generated using Glimmer3 [22] in an iterative fashion. The initial set of gene predictions was then used to train a second round of Glimmer3 analysis to produce the final set of gene predictions. All predicted genes were subsequently translated into all six reading frames and searched against a non-redundant amino-acid database using BLASTP. Each query protein-coding region was extended by 300 nucleotides in an attempt to extend the alignment through regions of low similarity and through different frames and stop codons using Blast-Extend-Repraze (BER, http://ber.sourceforge.net/). All putative protein coding sequences (CDS) were then searched against Pfam [23] and TIGRFAM [24] protein family models with HMMER3 [25]. Coding sequences that scored well to these models were assumed to share the function modeled by the HMM. All predicted proteins were then searched against the NCBI Protein Clusters Database (PRK) [26]. The remaining evidence types used in the automated functional annotation of gene products were SignalP [27], which detects the presence of putative signal sequences and TmHMM [28] to predict membrane-spanning regions.

The autoAnnotate program weighed the evidence obtained from the searches from a ranked list of evidence types to make a preliminary annotation, including name, gene symbol, Enzyme Commission (EC) [29] number, JCVI role category [30], and Gene Ontology (GO) [31] terms to each protein in the genome. Each protein was assigned a descriptive common name coming from an HMM name, a JCVI database of experimentally characterized proteins (CharProtDB) [32], or from a best BER match protein. Proteins predicted to encode enzymes were assigned EC numbers, JCVI role categories, GO terms and gene symbols (e.g., “*recA*”) as appropriate. The autoAnnotate program also employed the Protein Naming Utility (PNU) [33] to standardize protein nomenclature. Functional assignments were further enhanced with the Genome Properties [34] system, which records or predicts the presence or absence of metabolic pathways (e.g., biotin biosynthesis), protein complexes (e.g., ATP synthase), cellular structures (e.g., outer membrane) and certain genome traits (e.g., optimal growth temperature, cell shape, etc.). Additional structural features such as tRNAs were identified with the tRNAscanSE [35]. 16S and 23S ribosomal RNA genes were identified directly from BLAST search results. Other structural RNAs were identified from matches to Rfam, a database of non-coding RNA families [36] and Aragorn [37]. Insertion sequence elements were identified using the online tool ISsaga (http://issaga.biotoul.fr/ISsaga) with default settings [38]. Genomic islands (GIs) were identified using the online tool IslandViewer (http://www.pathogenomics.sfu.ca/islandviewer) [39], which integrates three different genomic island prediction methods: IslandPick [40], IslandPath-DIMOB [41], and SIGI-HMM [42]; we report putative GIs predicted by multiple tools.

### Comparative genomics

Regions of pairwise synteny between the *Leptospira* genomes were identified by first finding the maximum unique matches with a minimum length of five amino acids using PROmer [22], [43], followed by visualization of the data using MUMmerplot (http://mummer.sourceforge.net/) and Gnuplot 4.0 (http://www.gnuplot.info/) as previously described [44]. QuartetS [45] was used to identify orthologous protein sequences among the eight *Leptospira* genomes used in this study. QuartetS uses an approximate phylogenetic analysis of quartet gene trees to infer the occurrence of duplication events and discriminate paralogous from orthologous genes [45]. The QuartetS pipeline was run with default parameters. To be considered orthologs, the bi-directional best hit pairs had to satisfy the following conditions: (i) the alignment region had to cover at least 50% of the length of each sequence and (ii) the e-value of the pair-wise alignment had to exceed 1e^−5^.

To better understand the functional differences between pathogenic, intermediate and saprophytic *Leptospira*, each of the annotated genomes was uploaded to the RAST (**R**apid **A**nnotation using **S**ubsystem **T**echnology) server [46] retaining the original gene calls. Subsystems predicted to be active within each genome were then compared. A subsystem is a generalization of the concept of a biochemical pathway, extended to include ancillary components and alternative reactions reflecting functional variants found in various species.

### Prophage detection

Prophages were identified using *Phage_Finder* [47] version 2.0, which now utilizes HMMER3 [25], [48], drastically improving the speed of the HMM searches. Predicted prophage regions were identified using default settings and under strict (-S) mode. To facilitate identification of prophages in *Leptospira* genomes, Bacteriophage LE1 [49], [50] [51] was added to the BLAST database used for prophage identification. *Phage_Finder* version 2.0 is available at http://sourceforge.net/projects/phage-finder/files/phage_finder_v2.0/ under the GNU General Public License.

### Lipopolysaccharide preparation and gas chromatography mass spectrometery (GC-MS) analysis

A three-day culture of *L. licerasiae* str. VAR010 (∼10^8^ cells/mL) was harvested by centrifugation at 4000 rpm for 90 min at room temperature. Cells were washed thrice with 1× PBS then treated with 50% aqueous phenol for 30 min at 65°C with continuous stirring. The cells were immediately immersed in an ice-water bath to reduce the temperature to 10°C, then centrifuged at 4000 rpm for 40 min at 10°C. The top layer (phenol saturated aqueous layer) and bottom layer (water saturated phenol layer) were removed and dialyzed against ddH_2_O extensively to remove phenol (three days with change in water twice per day)—the phenol layer was analyzed by GC-MS and polyacrylamide gel electrophoresis. The dialyzed lipopolysaccharide (LPS) was lyophilized then re-suspended in 500 µL of water; 200 µL was used for sugar composition analysis. For GC-MS, samples were silylated using Trimethylsilyl (TMS). First, samples were methanolyzed using 1 M MeOH-HCl, at 80°C for 16 h, followed by re-N-acetylation and TMS derivatization using Tri-Sil TP reagent (Thermo Scientific) according to manufacturer's directions. The derivatives were subjected to GC-MS analysis and the data quantified using an internal inositol standard. LPS isolation and GC-MS analysis were done by the Glycotechnology Core Resource at the University of California, San Diego.

## Results and Discussion

### Assembly and annotation details of two draft *L. licerasiae* genomes

454 and Illumina pyrosequencing of str. VAR010^T^ yielded 2,272,294 reads that were assembled into 14 contigs (4 scaffolds) with 4,211,147 high-quality mostly contiguous bases. These contigs had an average length of 300.8 kb, an N50 of 522.9 kb and a maximum length of 1.67 mb. The str. MMD0835 genome was assembled into 48 contigs with 4,198,811 contiguous bases (N50 of 463.5 kb; max. length of 1.07 mb). The overall characteristics of the draft *L. licerasiae* genomes are summarized in Table 1. G+C content. Gaps in genome coverage were not filled in with manual sequencing given resource constraints. This approach is consistent with *de novo* sequencing and publication of other pathogen genomes, given that the length of the draft genomes was consistent with other sequenced leptospiral genomes (Table 1) and that the two strains whose genome sequences reported here are vastly similar. Gaps are typically caused by large (greater than the library “insert” size) fragments, which tend to be rRNA operons, large mobile elements or duplicated regions and likely do not materially detract from the quality of the data analysis presented here.

**Table 1:** Salient features of the unfinished *L. licerasiae* genomes.

	*L. licerasiae* Varillal VAR010*	*L. licerasiae* Varillal MMD0835*	*L. interrogans* Copenhageni Fiocruz‡	*L. interrogans* Lai 56601‡	*L. borgpeterseni* Hardjo L550‡	*L. borgpetersenii* Hardjo JB197‡	*L. biflexa* Patoc Ames‡	*L. biflexa* Patoc Paris‡
Size (Mbp)	∼4.2	∼4.2	∼4.6	∼4.7	∼3.9	∼3.9	∼4.0	∼4.0
G+C (%)	41.6	41.1	35.1	36.0	40.2	40.2	38.6	38.9
CDS								
Hypothetical proteins	1302	1600	1833	1579	963	949	1221	1532
Proteins with functional assignments	2629	2285	1834	2123	1982	1931	2379	2194
Total	3931	3885	3667	3702	2945	2880	3600	3726
tRNA genes	37	37	37	37	37	37	35	35
Riboswitches and *cis*-regulatory elements								
Cobalamin	2	2	2	2	2	2	NP^b^	NP
TPP	Y^c^	Y	Y	Y	Y	Y	Y	Y
*cis*-element	Y [*ydaO-yuaA*]	Y [*ydaO-yuaA*]	NP	NP	NP	NP	NP	NP
CRISPR's (# repeats)	NP	NP	Y(4)	Y(18)	NP	NP	NP	NP
rRNA								
23S	1	1	2	2	2	2	2	3
16S	1	1	2	2	2	2	2	2
5S	2	2	1	1	1	1	2	2
N50/kb^a^	522.9	463.5	NA^d^	NA	NA	NA	NA	NA
Species-specific genes	1211		649		225		1553	

* Draft Genome.

‡ Genome version as of July 01, 2011—includes all replicons and plasmids.

a N50 = length-weighted mean: the size of the smallest contig such that 50% of the genome is contained in contigs of size N50 or greater.

b NP – Not Present.

c Y – Yes.

d NA – Not Applicable.

### General genome features of *L. licerasiae* str. VAR010 and MMD0835

#### Non-coding RNA (ncRNA) genes and regulatory elements

The *L. licerasiae* genomes were examined for the presence of riboswitches [52] and other ncRNA regulatory elements. Riboswitch predictions in the finished leptospiral genomes were confirmed by an online search of the Rfam database [36]. Only candidates passing Rfam trusted cutoffs and therefore very likely to be true ncRNAs are presented. All infectious *Leptospira* contain at least two copies of the cobalamin (vitamin B_12_) riboswitch. As in other bacteria, both riboswitches appear to regulate expression of genes necessary for transport and biosynthesis of vitamin B_12_. The first, LEP1GSC185_0331, is immediately upstream of a gene encoding a TonB-dependent ligand-gated channel with similarity to the outer membrane cobalamin transport protein, BtuB, and the second, LEP1GSC185_3336, is immediately upstream of two genes encoding a putative cobalt transporter (*cbtA*—LEP1GSC185_3338; LlicsVM_010100017167 and *cbtB*—LEP1GSC185_3337; LlicsVM_010100017162) and the adjacent cobalamin biosynthesis (*cob*) operon. The lack of a cobalamin riboswitch and an incomplete *cob* operon in the saprophyte *L. biflexa* (see below) suggest that the ability to respond to cobalamin levels and synthesize B_12_ *de novo* from simpler metabolites is restricted to infectious *Leptospira*. Interestingly, the *L. licerasiae* genes encoding CbtA and CbtB, which share homology with *Pseudomonas syringae* proteins, may have been acquired via lateral gene transfer (LGT) since these genes are uncommon in *Leptospira*—homologs of both proteins are also present in *L. broomii*, *L. inadai* and *L. kmetyi*. All of the genomes studied here possess a single thiamine pyrophosphate (TPP; LEP1GSC185_0557) riboswitch (Table 1) that in *L. licerasiae* is directly upstream of *thiC* (LEP1GSC185_0556). The thiamine biosynthesis protein, ThiC, converts 5′-phosphoribosyl-5-aminoimidazole to 4-amino-5-hydroxymethyl-2-methylpyrimidine, an important intermediate in the synthesis of TPP.

A putative *cis*-regulatory element unique to *L. licerasiae* was also identified, *ydaO*-*yuaA* (LEP1GSC185_1591). This element is thought to be triggered during osmotic shock leading to activation of *ydaO*, a predicted amino acid transporter gene, and members of *yuaA*-*yubG* operon, which encode KtrA and KtrB K^+^ transporters [53]. While a role in *L. licerasiae* is yet to be established, this element is found immediately upstream of a universal stress family protein (LEP1GSC185_1590; LlicsVM_010100003660), which has homology at the C-terminus to a family of universal stress proteins (USPs) and Na+/H+ exchangers (NHEs). USPs are small cytoplasmic bacterial proteins whose expression increases when the cell is exposed to stress agents such as DNA-damaging agents [54]. These proteins are thought to enhance survival during prolonged exposure to such conditions [54]. Indeed, one such protein UspA is up regulated in *Leptospira* at physiological temperature implying a role during *in vivo* growth [55]. NHEs are found in both prokaryotes and eukaryotes and are believed to be crucial for cell volume homeostasis [56]. Thus, it is possible that in *L. licerasiae*, the *ydaO*-*yuaA* element responds to and permits survival during periods of osmotic stress. This mechanism could allow for survival in environmental waters.

### Prophages

Prophages can be important drivers of microbial evolution by providing fitness factors for their host [57], [58], by facilitating movement of DNA through transduction of the host chromosome or packaging of pathogenicity islands [59] and altering serotype through lysogenic conversion [60], [61]. To explore any of these possibilities in any of the available *Leptospira* genomes, *Phage_Finder* [47] was run under strict (-S) mode to identify prophage regions. *Phage_Finder* identified two prophage regions in the genomes of both *L. licerasiae* strains.

**Figure 1:**
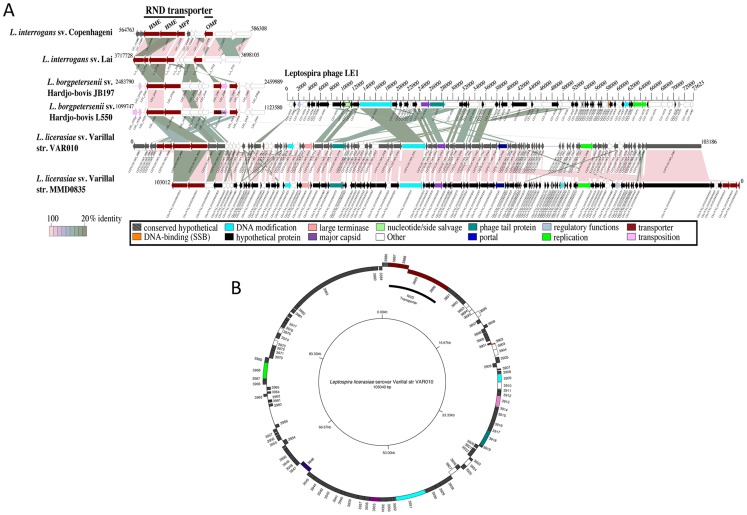
Comparison of LE1-like prophage regions in *L. licerasiae*. (A) Linear representations of CDSs found in each *L. licerasiae* genome with similarity to bacteriophage LE1 and to non-prophage regions of *L. interrogans* and *L. borgpetersenii* encoding efflux pumps. CDSs are labeled by locus identifier and colored by functional role categories as noted in the boxed key. BLASTP matches between CDSs are colored by protein percent identity (see key). (B) Circular depiction of vB-LliZ_VAR010-LE1.

The first region in each strain was located on ∼103 kb contigs (AHOO02000007 in VAR010 and NZ_AFLO01000023 in MMD0835) with best BLASTP matches to bacteriophage LE1 of *L. biflexa*. LE1 was previously shown to be of circular topology, to form intracellular particles consistent with phage, and to replicate like a plasmid [51]. Given this information and that a large portion of each contig was predicted to be prophage, it was reasonable to believe these phage-like contigs in *L. licerasiae* also represented linear forms of circular phage genomes like LE1. There was significant overlap in the sequence of the ends, also suggesting a circular form. To demonstrate circular topology, outward-facing primers were designed and used in PCR reactions. The results of PCR produced 300 bp products, indicating that both LE1-like phages are indeed circular in *L. licerasiae* strains VAR010 and MMD0835 (Figure 1). Comparisons between LE1 and these prophages at the protein level indicated that the later three quarters of the *L. licerasiae* prophage proteins match some portion of LE1 (Figure 1), albeit at a low percent identity (average ∼30% identity). A comparison between the two LE1-like prophages revealed that they are identical at the amino acid level (Figure 1). We propose naming the *L. licerasiae* LE1-like prophages vB-LliZ_VAR010-LE1 and vB-LliZ_MMD0835-LE1 using a previously suggested systematic bacteriophage nomenclature [62]. vB-LliZ_VAR010-LE1 encodes 102 predicted proteins and has a G+C of 37.8% which is lower than the average for the entire *L. licerasiae* genome—41.6%. These *L. licerasiae* prophage elements possess ∼22 kb of unique sequence that LE1 lacks as well as several unique predicted open reading frames interspersed among the LE1 homologs. A comparison of this ∼22 kb region to other *Leptospira* genomes identified multiple efflux pumps in the infectious *L. licerasiae* that may function in adaptation to the mammalian host. Further, this amino acid similarity to bacterial efflux pumps suggests phage-mediated gene transfer between the *L. licerasiae* chromosome and LE1-like prophage. While the presence of these efflux pumps in the genomes of other infectious species would also suggest a role in pathogenicity, BLASTP searches against the non-redundant protein database (nr) indicate that these proteins have homologs in the non-pathogen *Leptonema illini* DSM 21528. Why *L. licerasiae* and not *L. biflexa* have maintained copies of these genes is unclear. Also within this region, the predicted *L. licerasiae* protein LEP1GSC185_3887 is notable in that it shares homology with a TolC/IS1533 transposase fusion protein. It has been suggested that the mobile genetic element (MGE) IS1533, has mediated LGT resulting in the antigenic switch of sv. Copenhageni to sv. Hardjo [63].

**Figure 2:**
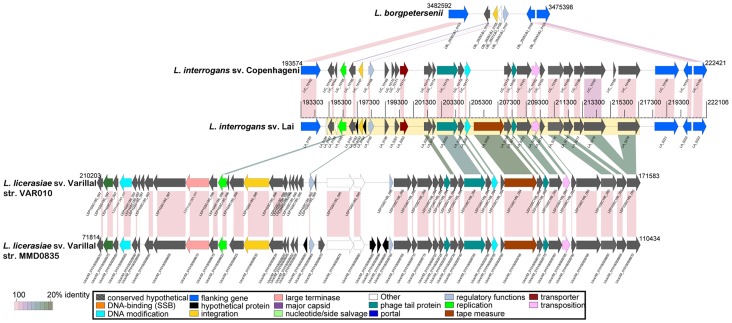
Comparison of prophage regions in *L. licerasiae* similar to a known cryptic prophage in expressed in *L. interrogans* sv. Lai. Depicted are linear representations of CDSs found in each genome with similarity to the cryptic prophage identified by Qin et al. in *L. interrogans* sv. Lai [64]. CDSs are labeled by locus identifier and are colored by functional role categories as noted in the boxed key. BLASTP matches between CDSs are colored by protein percent identity (see key). CDSs colored blue in *L. borgpetersenii* and *L. interrogans* genomes denote CDSs flanking the regions identified by Qin et al. (yellow highlighted box).

The second prophage region was only partially detected in the *L. licerasiae* genomes by *Phage_Finder* (Figure 2), but is adjacent to a cryptic prophage region expressed in *L. interrogans* sv. Lai and is presumably associated with pathogenicity [64]. The region detected by Phage_Finder is located at nucleotide position 210203..191954 of VAR010 and 71814..108770 of MMD0835, but after comparison to the above mentioned unnamed prophage element in *L. interrogans* sv. Lai, could be extended to include coordinates 210203..171583 of VAR010 and 71814..110434 of MMD0835 (Figure 2). Presumably the reason this region was truncated by *Phage_Finder* was due to a lack of sufficient homology in the BLAST database used and/or due to the lack of a head morphogenesis region, which is required by *Phage_Finder* to label a region as “prophage” under strict mode. Since this region lacks an identifiable head morphogenesis region yet retains tail-like proteins, it may be functionally analogous to phage tail-type bacteriocins, called pyocins in *Pseudomonas aeruginosa* [65] and monocins in *Listeria* [66].

### Comparison of the pathogenic, intermediately pathogenic and saprophytic leptospiral genomes

*L. licerasiae* str. VAR010 causes mild disease in humans and has been isolated from peridomestic and wild rodents and marsupials in Peru [8]. Although phenotypic differences between VAR010 and MMD0835 have yet to be described, VAR010 (3931 total CDS) has 185 non-orthologous CDS relative to strain MMD0835 (3885 total CDS), whereas strain MMD0835 has 140 non-orthologous CDS relative to strain VAR010 reminiscent of another environmental pathogen with a plastic genome, *Burkholderia pseudomallei* [67]. The majority of these non-orthologous genes encode hypothetical proteins. Both strains share 3,745 CDS with an average pair-wise amino acid similarity of 99.98%. Of these, 1211 have no orthologs in the other genomes used in this study. A putative function could be assigned to 632 with the remainder comprising hypothetical (579) proteins (Table S1).

**Figure 3:**
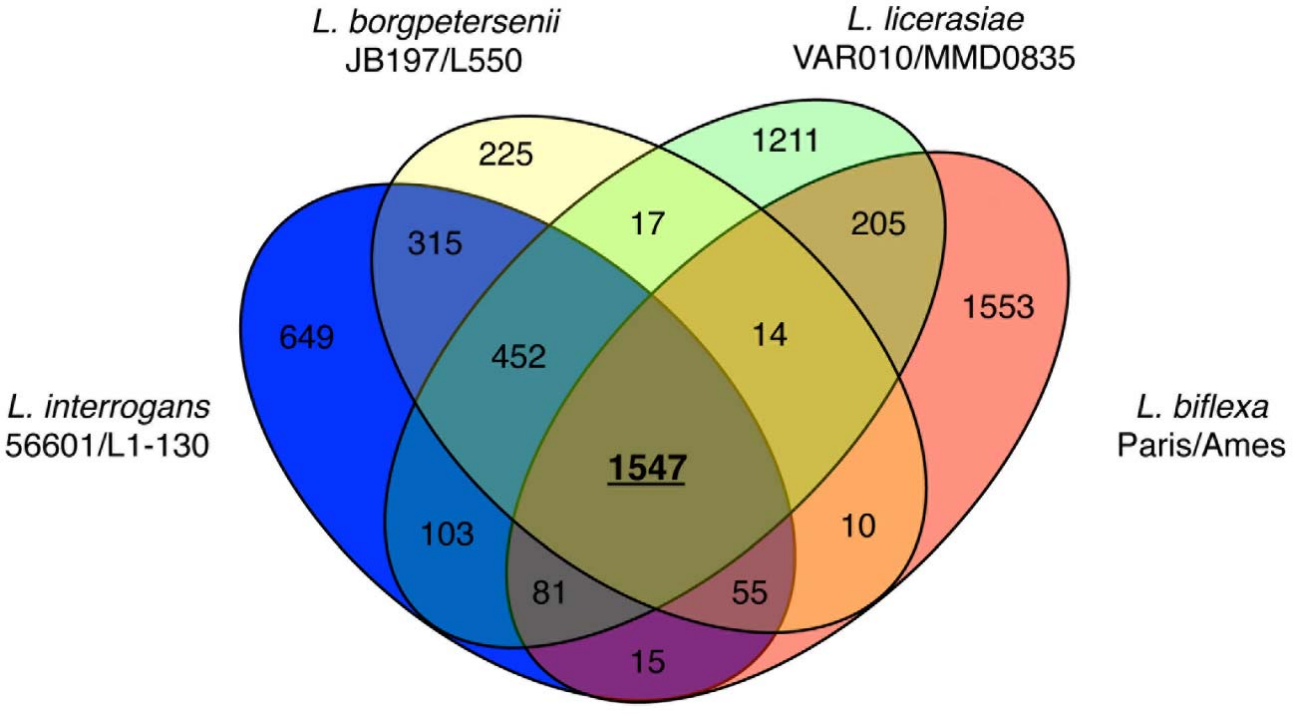
Venn diagram showing the distribution of 3745 *L. licerasiae* orthologs by leptospiral species. Orthologs were predicted using QuartetS [45] run with default parameters. Only proteins present in both strains of a given species are shown.

**Figure 4:**
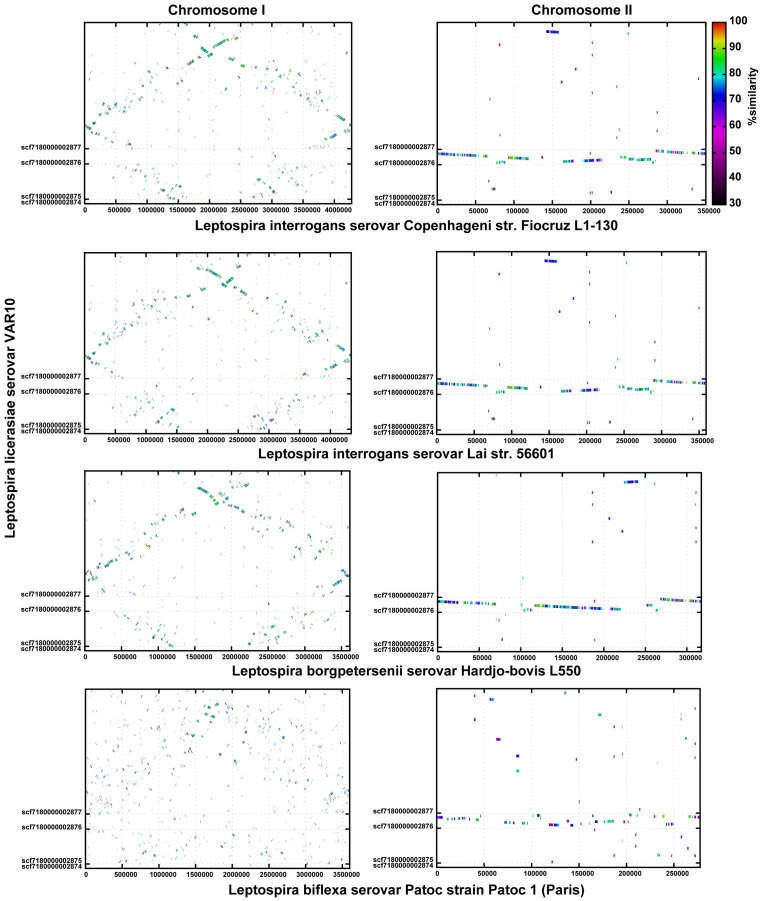
Whole-genome comparison of the pathogenic, intermediately pathogenic and saprophytic species of *Leptospira*. Line figures depict the results of PROmer analysis. Colored lines denote percent identity of protein translations (see key) and are plotted according to the location in the leptospiral reference genomes (x-axis) and the query genome *L. licerasiae* strain VAR10 (y-axis). Chromosome I results are in the left column while chromosome II comparisons are in the right column.

Considering only those genes common to both strains of each species, *L. licerasiae* shares 2,237 (∼57%) with *L. interrogans*, 2,077 (∼53%) with *L. borgpetersenii* and 1,898 (∼48%) with *L. biflexa*. 1,547 orthologs (∼39% of the predicted *L. licerasiae* CDS) were present in all genomes compared (Figure 3) and likely represent a substantial proportion of the core genome of *Leptospira*. As shown in Figure 4, the gene order is more conserved in the intermediate and pathogenic branches. Surprisingly, *L. licerasiae* had the highest average protein identity with *L. interrogans* sv. Lai (2,278 proteins with an average pairwise identity of ∼67%). Taken together these observations suggest that *L. licerasiae* is more closely related to the pathogenic branch of infectious *Leptospira* than to the saprophyte, *L. biflexa*. This was unexpected since 16S rRNA phylogeny suggests that *L. licerasiae* occupies an intermediate position between the pathogens and saprophytes [8]. Table 2 shows the subsystem distribution of predicted CDS in *L. licerasiae*, *L. interrogans*, *L. borgpetersenii* and *L. biflexa*. Based on these data it would seem that intermediate *Leptospira* retain several proteins related to nitrogen, amino acid and carbohydrate metabolism that have likely been lost by the pathogenic sub-branch. For example, *L. licerasiae* (LEP1GSC185_2652) and *L. biflexa* (LEPBI_I1590) both possess *ilvA*, which encodes threonine ammonia-lyase an enzyme that catalyzes the conversion of threonine to 2-oxobutanoate; while neither *L. borgpetersenii* nor *L. interrogans* appears to do so. That *L. licerasiae* and perhaps the other intermediates do well in artificial culture media might be related to the retention of these metabolic functions.

**Table 2:** Distribution of subsystems in *Leptospira*. Predicted by RAST server.

Subsystem	VAR010	MMD0835	L1–130	56601	L550	JB197	Paris	Ames
Cofactor, Vitamins, Prosthetic Groups, Pigments	156	155	202	201	137	138	141	141
Cell Wall and Capsule	71	71	100	110	94	94	79	79
Virulence, Disease and Defense	55	55	40	37	34	33	62	61
Potassium Metabolism	13	13	18	18	9	9	0	0
Miscellaneous	99	99	145	143	95	94	96	96
Membrane Transport	25	26	19	24	11	10	14	28
Iron acquisition and metabolism	8	8	2	2	1	1	6	6
RNA metabolism	107	96	121	116	95	92	105	105
Nucleotides and Nucleosides	50	50	55	55	49	48	51	51
Protein Metabolism	185	185	184	182	204	188	154	182
Cell Division and Cell Cycle	22	20	24	23	20	20	21	21
Motility and Chemotaxis	82	82	75	78	81	81	106	104
Regulation and Cell Signaling	43	40	17	51	20	19	61	64
Secondary Metabolism	4	4	4	4	4	4	4	4
DNA Metabolism	68	77	63	59	50	49	73	73
Fatty Acids, Lipids and Isoprenoids	73	72	77	78	58	45	63	46
Nitrogen Metabolism	**22**	**22**	4	5	5	4	**24**	**24**
Dormancy and Sporulation	3	3	3	3	3	3	4	3
Respiration	64	64	51	52	50	50	64	64
Stress Response	75	75	60	62	57	55	86	86
Metabolism of Aromatic Compounds	3	3	1	1	1	1	1	1
Amino Acids and Derivatives	**219**	**219**	180	176	172	171	**216**	**216**
Sulfur Metabolism	8	20	19	18	17	17	12	12
Phosphorous Metabolism	**22**	**22**	14	13	11	12	**19**	**19**
Carbohydrates	**179**	**179**	102	107	130	131	**176**	**164**

It is a commonly accepted concept that genes unique to pathogenic microorganisms are likely to be necessary for infection (pathogenesis). To identify potentially pathogenicity-associated genes, we compared the genome content of three infectious leptospiral species, *L. licerasiae* (2 strains), *L. interrogans* (2 strains) and *L. borgpetersenii* (2 strains) with that of the non-infectious saprophyte, *L. biflexa* (2 strains). These comparisons identified 452 conserved pathogen-specific proteins (Figure 3). Based on domain homology searches, 315 were assigned a putative function (Table S2). Infectious *Leptospira* species share a number of proteins predicted to participate in environmental signaling and processing and metabolism (Table S2).

That the infectious species studied here appear to possess a complete vitamin B_12_ biosynthesis operon and a novel regulatory mechanism is perhaps the most notable metabolic difference between infectious and non-infectious *Leptospira*. Indeed the absence of these genes from the *L. biflexa* and recently sequenced *Leptonema* genomes would indicate that the ability to synthesize B_12_ was acquired after the speciation event giving rise to the infectious branch of *Leptospira* predating the separation of the intermediate and pathogenic sub-branches. The genomes of over 100 infectious strains searched so far including the intermediates species, *L. inadai* and *L. broomii*, possess at least two copies of the B_12_ riboswitch (M. Matthias and J. Vinetz manuscript in preparation), supporting the belief that these elements are essential for pathogenicity. As in other bacteria, the availability of different nutrients inside and outside the mammalian host requires changes in the metabolic capacity of *Leptospira*. Published data have firmly established that *Leptospira* have an absolute requirement for B_12_ for growth at 37°C [68]. Much like iron, B_12_ is sequestered *in vivo*. Hence, for survival *in vivo*, leptospiral pathogens need to synthesize B_12_ *de novo* or scavenge B_12_ from the host. Whether leptospires are fully capable of synthesizing the highly complex B_12_ molecule from simpler precursors *de novo* is not known. But, *cobI* (LEP1GSC185_3345; LIC20129), an enzyme involved in cobalamin biosynthesis, is ∼30-fold up regulated during mammalian infection consistent with a role *in vivo* in replication and/or pathogenicity (J. Lehmann, J. Vinetz, and M. Matthias manuscript in preparation). In addition, although all leptospiral genomes sequenced to date, including *L. biflexa*, encode the enzyme cob(I)yrinic acid a,c-diamide adenosyltransferase, which catalyzes the first step in the conversion of cobinamide to B_12_, all infectious *Leptospira*, including *L. licerasiae*, *L. interrogans*, *L. borgpetersenii*, *L. santarosai*, *L. noguchii* and *L. weilii*, encode at least one additional homolog. The reason for this is unclear, but it may be that these pathogen-specific homologs are required for B_12_ biosynthesis *in vivo*. While *L. interrogans*, *L. borgpetersenii* and *L. licerasiae*, appear to be able to use either l-glutamate or cobinamide to synthesize B_12_, it would seem that this is not a universal feature of infectious *Leptospira*.

*Leptospira* encode four essential B_12_-dependent enzymes: B_12_-dependent methionine synthase, two B_12_-dependent methylmalony-CoA mutase related proteins and a B_12_-dependent ribonucleotide reductase. Methionine synthase transfers a methyl group from methyl-tetrahydrofolate to homocysteine as the final step in the synthesis of methionine; ribonucleotide reductases generate the deoxyribonucleotides needed for DNA synthesis and allow the production of DNA in the absence of oxygen; methylmalonyl-CoA interconverts (R)-methylmalonyl-CoA and succinyl-CoA in the terminal step of *β*-oxidation of fatty acids/catabolism of cholesterol. A role for B_12_ in leptospiral pathogenicity has yet to be established. However, B_12_ synthesis has been linked to fatty acid metabolism and survival of the intracellular pathogen *Mycobacterium tuberculosis in vivo* [69]. As humans do not synthesize B_12_, these genes may represent novel drug targets.

### Putative pathogenicity-associated genes

The *L. licerasiae* VAR010 and MMD0835 genomes encode 196 and 198 putative lipoproteins, respectively consistent with the number found in other leptospiral species (*L. interrogans* – 184; *L. borgpetersenii* – 130 and *L. biflexa* 164) [20]. Of these, infectious species share LipL31 (LEP1GSC185_3242, LlicsVM_010100016712), LipL32 (LEP1GSC185_2633, LlicsVM_010100013757), LipL40 (LEP1GSC185_1670, LlicsVM_010100003275), LipL41 (LEP1GSC185_1838, LlicsVM_010100002470), LipL46 (LEP1GSC185_3176, LlicsVM_010100016407), LigB (LEP1GSC185_1828; LlicsVM_010100002515), LruA/LipL71 (LEP1GSC185_0209, LlicsVM_010100006058) and LruB (LEP1GSC185_0754, LlicsVM_010100019404). That these genes are absent from the *L. biflexa* genome suggests a potential role in pathogenicity. The function of LruB is unknown, but serology suggests this protein is expressed *in vivo* [70].

Much recent work has demonstrated the importance of fibronectin and plasminogen binding proteins in *Leptospira* [71]–[82]. Fibronectin binding proteins are adhesins that play an important role in certain bacterial infections [83], [84]. Putative pathogenicity factors LigA and LigB, specific to pathogenic *Leptospira* are induced at physiological osmolarity and are involved in leptospiral adhesion to extracellular matrix proteins and plasma proteins including collagens I and IV, laminin, fibronectin and fibrinogen [85]. The above mentioned LipL32 and LipL40 are putative plasminogen binding proteins [81]. Apart from LigB, at least three other conserved pathogen-specific outer membrane proteins are predicted to mediate attachment to host cells: a putative fibronectin binding protein, Lfb1 (LEP1GSC185_0134; LlicsVM_010100012092) [86]; Lsa66, a leptospiral surface adhesin of 66 kDa (LEP1GSC185_1758; LlicsVM_010100002865) shown to bind laminin and plasma fibronectin extracellular matrix molecules [72], and a protein believed to mediate attachment to host cells (LEP1GSC185_2102; LlicsVM_010100001165).

### Unique genomic features of the *L. licerasiae* O-antigen locus

**Figure 5:**
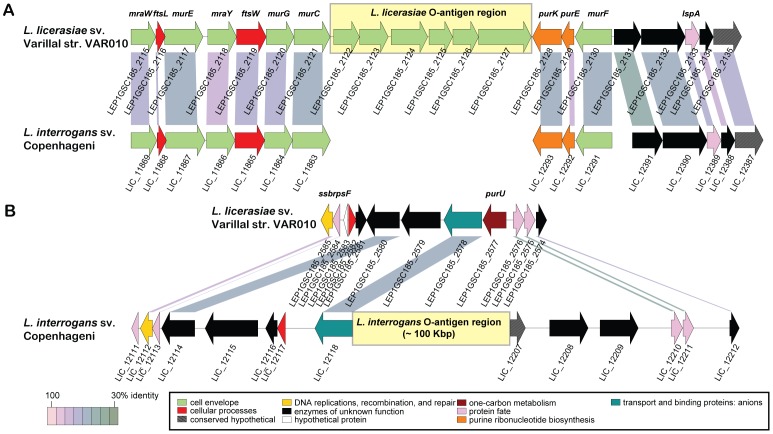
Structure of *Leptospira* O-antigen regions. The O-antigen region and flanking CDSs of *L. licerasiae* strain VAR10 are compared to regions of homology in *L. interrogans* Copenhageni (A). Likewise, the O-antigen and flanking CDSs of *L. interrogans* Copenhageni are compared to the homologous region in *L. licerasiae* strain VAR10 (B). Yellow shaded boxes mark the locations of the O-antigen regions. CDSs are labeled by locus identifier and colored by functional role categories as noted in the boxed key. Gene symbols, when present, are noted above their respective genes in bold italics. BLASTP matches between CDSs are colored by protein percent identity (see key).

Previously published immunological data from Peru indicate that the *L. licerasiae* O-antigen is antigenically unique [8]. Comparative analysis of all extant *Leptospira* spp. genomic data, including the new data presented here, explains this antigenic uniqueness at a genomic level. In contrast to the complex LPS O-antigen biosynthetic loci found in the published *L. interrogans*, *L. borgpetersenii* and *L. biflexa* genomes, which contain 91, 76 and 56 genes respectively, the *L. licerasiae* O-antigen locus we propose is comprised of a modest 6-gene operon, LEP1GSC185_2122–2127 (Figure 5, Table 3). The genes in this cluster have no apparent orthologs in the already sequenced *L. interrogans*, *L. borgpetersenii* and *L. biflexa* genomes. We are confident that this operon is the true *L. licerasiae* O-antigen locus based on the following observations: 1) There are only two *wzx* O-antigen transporter homologs in the genome. One of these (LEP1GSC185_0029) is not in an operon with any other genes of types typically associated with O-antigen biosynthesis. The other, LEP1GSC185_2124, is part of the proposed O-antigen locus. 2) Of the 29 putative polysaccharide glycosyltransferases we could identify in the *L. licerasiae* genome, while none are orthologs of genes in the O-antigen regions of the other sequenced *Leptospira* genomes, 22 are bidirectional best hits (that is, candidate orthologs) with non-O-antigen related genes from one or more of these genomes. Of the remaining 7 genes, one (LEP1GSC185_3401) is associated with a glycogen-related operon, two (LEP1GSC185_1696 and _2304) are part of short operons with genes of unknown function, and one (LEP1GSC185_2985) is proximal to flagellin genes. The remaining three, LEP1GSC185_2122, 2123 and 2126, are clustered together in the proposed O-antigen locus. 3) The remaining two genes in the proposed O-antigen locus, LEP1GSC185_2125 and 2127, encode functions commonly associated with sugar modification in O-antigens, pyruvoylation and acetylation, respectively. Homologs of the latter gene are specifically annotated as O-antigen related. 4) This O-antigen region is fully within a single contig.

**Table 3:** The top 10 BLAST hits from the six genes of the proposed *L. licerasiae* O-antigen locus identify genes from sixty unique genomes.

*L. licerasiae* str. VAR010	LEP1GSC185_2122	LEP1GSC185_2123	LEP1GSC185_2124	LEP1GSC185_2125	LEP1GSC185_2126	LEP1GSC185_2127
Annotation	glycosyltransferase group 2 family protein	glycosyltransferase group 1 family protein	O-antigen transporter (flippase), wzx	polysaccharide pyruvoyl transferase domain protein	glycosyltransferase group 1 family protein	O-antigen acetylase
*L. licerasiae* str. MMD0835	LlicsVM_010100001060 ZP_09256931	LlicsVM_010100001055 ZP_09256930	LlicsVM_010100001050 ZP_09256929	LlicsVM_010100001045 ZP_09256928	LlicsVM_010100001040 ZP_09256927	LlicsVM_010100001035 ZP_09256926
Best BLAST hits	*Xylanimonas cellulosilytica* DSM 15894 (ACZ31569) E = 6e-100	*Methanococcus maripaludis* C6 (ABX01399) E = 2e-71	Flavobacteriaceae bacterium HMQ9 (ZP_09314923) E = 6e-66	*Leeuwenhoekiella blandensis* MED217 (EAQ47946) E = 7e-83	*Thermus thermophilus* SGO.5JP17-16 (AEG33390) E = 2e-05	*Xylella fastidiosa* 9a5c (AAF83588) E = 2e-170
	Nocardioidaceae bacterium Broad-1 (EGD43512) E = 6e-96	*Desulfovibrio desulfuricans* ND132 (EGB14691) E = 7e-70	*Clostridium symbiosum* WAL-14673 (EGB20289) E = 1e-65	Flavobacteriaceae bacterium S85 (ZP_09498960) E = 8e-54	*Pseudomonas syringae* pv. syringae B728a (AAY35972) E = 3e-05	*Metylomonas methanica* MC09 (AEG01963) E = 2e-163
	marine actinobacterium PHSC20C1 (EAR24699) E = 2e-95	Rhodobacteraceae bacterium KLH11 (EEE35994) E = 2e-65	*Bacillus coagulans* 36D1 (AEP00093) E = 3e-65	*Lacinutrix* sp. 5H-3-7-4 (AEH00078) E = 8e-53	*Pseudomonas syringae* pv. japonica str. M301072PT (EGH31218) E = 1e-04	*Methylocella silvestris* BL2 (ACK52753) E = 2e-148
	*Isoptericola variabilis* 225 (AEG45117) E = 9e-94	*Herpetosiphon aurantiacus* DSM 785 (ABX05989) E = 5e-65	*Clostridium* sp. 7_3_54FAA (EHF07292) E = 1e-57	*Arthrospira maxima* CS-328 (EDZ95273) E = 3e-38	uncultured methanogenic archaeon RC-I (CAJ36730) E = 2e-04	*Methylotenera versatilis* 301 (ADI30623) E = 9e-142
	candidate division TM7 genomospecies GTL1 (EDK72664) E = 7e-92	*Solitalea canadiensis* DSM 3403 (AFD06051) E = 8e-62	*Desulfosporosinus meridiei* DSM 13257 (EHC17232) E = 6e-56	*Methylovorus glucosetrophus* (ACT51074) E = 9e-38	*Streptococcus macacae* NCTC 11558 (EHJ51543) E = 9e-04	*Herbaspirillum seropedicae* SmR1 (ADJ62198) E = 8e-137
	*Alteromonas macleodii* str. ‘Deep ecotype’ (AEA97644) E = 9e-92	*Chloroflexus aurantiacus* J-10-fl (ABY35134) E = 1e-60	*Thermosipho africanus* TCF52B (ACJ75209) E = 7e-54	*Synechococcus* sp. PCC 7335 (EDX85506) E = 6e-37	*Clostridium botulinum* B str. Eklund 17B (ACD22596) E = 0.001	marine gamma proteobacterium HTCC2148 (EEB79327) E = 2e-135
	*Nitrosococcus halophilus* Nc4 (ADE13571) E = 1e-91	*Fluviicola taffensis* DSM16323 (AEA45998) E = 2e-59	*Chryseobacterium gleum* ATCC35910 (EFK35853) E = 4e-53	*Rhodanobacter* sp. 2APBS1 (EHA64969) E = 3e-35	*Methanosphaerula palustris* E1-9c (ACL17229) E = 0.002	*Azoarcus* sp. BH72 (CAL96196) E = 9e-135
	*Cellulomonas flavigena* DSM 20109 (ADG75250) E = 4e-91	*Chloroflexus aggregans* DSM 9485 (ACL25440) E = 3e-59	*Dorea formicigenarans* 4_6_53AFAA (EGX71055) E = 3e-51	*Rhizobium leguminosarum* bv. trifolii WSM1325 (ACS57464) E = 2e-34	*Pseudomonas syringae* pv. aptata str. DSM 50252 (EGH75603) E = 0.003	*Herbaspirillum seropedicae* SmR1 (ADJ63706) E = 4e-130
	Verrucomicrobiae bacterium DG1235 (EDY81368) E = 9e-91	*Methanococcus aeolicus* Nankai-3 (ABR55981) E = 4e-59	butyrate-producing bacterium SS3/4 (CBL39981) E = 5e-51	*Rhizobium etli* 8C-3 (ZP_03513426) E = 3e-34	*Pseudomonas syringae* pv. morsprunum str. M302280PT (EGH07487) E = 0.004	*Pseudomonas aeruginosa* 138244 (EGM19323) E = 1e-128
	*Clostridium* sp. D5 (EGB90754) E = 1e-90	*Roseiflexus* sp. RS-1 (ABQ89511) E = 1e-58	*Eubacterium dolichum* DSM 3991 (EDP10489) E = 1e-49	*Methylobacter tundripaludum* SV96 (EGW21083) E = 3e-34	*Vibrio caralliilyticus* ATCC BAA-450 (EEX30732) E = 0.005	*Herbaspirillum seropedicae* SmR1 (ADJ63498) E = 1e-124

The boundaries of the proposed *L. licerasiae* O-antigen biosynthesis operon map to three different syntenic regions in *L. interrogans*, which suggests a complex history of differential genome rearrangement and LGT events in these two species. Indeed, the six genes of the operon do not correspond to any syntenic blocks in any sequenced genome – the most similar genes to each are present in entirely disjoint sets of bacterial and archaeal strains (Table 3). This would seem to imply either, 1) that potential *en bloc* LGT source genomes with similarly constructed O-antigens have yet to be sequenced, or 2) that a series of LGT events from different sources have accumulated these genes in the *L. licerasiae* lineage to create a novel O-antigen cluster. Since the O-antigen cluster is not predicted to reside on a GI the latter seems more likely.

By analogy to extant knowledge of how *E. coli* O-antigen operons are assembled [87], we can hypothesize that the *L. licerasiae* O-antigen consists of a repeating unit with at least 4 sugars (corresponding to the primer sugar and the products of the three glycosyltransferase enzymes), that these sugars are bioavailable from the core *Leptospira* metabolome (i.e. glucose, galactose, mannose, etc., since no blocks of sugar biosynthesis or sugar modification genes are present in the operon) and at least one of them is modified by (probably) pyruvoyl and/or acetyl groups.

**Figure 6:**
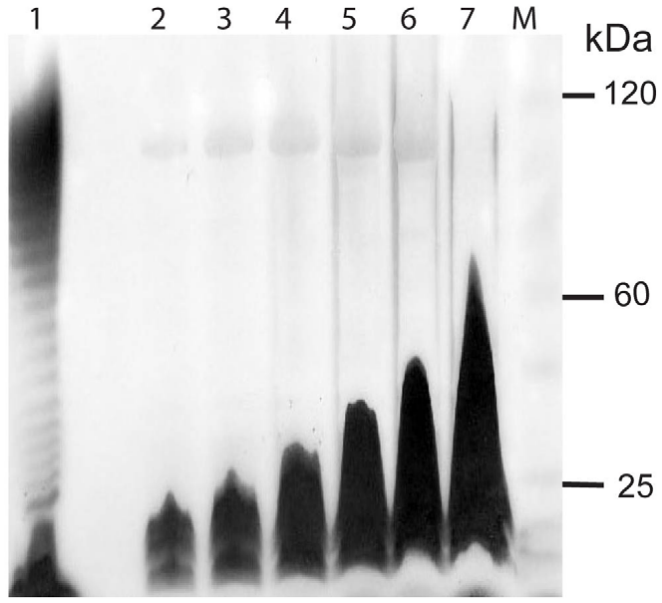
Silver-stained polyacrylamide gel of purified *L. licerasiae* str. VAR10 LPS. LPS was purified by the hot-phenol water method. *E. coli* LPS (Lane 1), 1/32 Dilution of VAR010 Phenol phase LPS (Lane 2), 1/16 Dilution of VAR010 Phenol phase LPS (Lane 3), 1/8 Dilution of VAR010 Phenol phase LPS (Lane 4), 1/4 Dilution of VAR010 Phenol phase LPS (Lane 5), 1/2 Dilution of VAR010 Phenol phase LPS (Lane 6), Undiluted VAR010 Phenol phase LPS (Lane 7), Molecular weight marker (M).

The chemical composition of the polysaccharide component of leptospiral LPS has been examined in a few serovars [88]–[90]. The proportion of the major component sugars rhamnose, galactose, arabinose and xylose was shown to vary between strains. The composition of the LPS derived from *L. licerasiae* sv. Varillal is consistent with previously published data. However, our GC-MS analysis indicates that *L. licerasiae* LPS (Figure 6) is composed primarily of arabinose (∼61.6%), with xylose (∼12.8%), mannose (∼11.5%), rhamnose (∼9.3%), galactose (∼4.0%) and glucose (<1%). The relative proportion of arabinose and rhamnose (6∶1) in the LPS of *L. licerasiae* is significantly different from that (1∶3) reported in *L. interrogans* sv. Copenhageni [89], which might help to explain why there is absolutely no serological cross-reactivity between sv. Varillal and Copenhageni [8]. The presence of rhamnose in the purified VAR010 LPS is surprising since the genome does not appear to encode a complete pathway for the synthesis of dTDP-l-rhamnose shown to be present in *L. interrogans* and *L. borgpetersenii*; the enzymes that catalyze the final two steps on the pathway, *rmlC* and *rmlD*, are absent. Because both *L. licerasiae* genomes are unfinished, it is possible that these genes reside on unsequenced regions of the genome. But, since other intermediate strains sequenced to date, *L. broomii* and *L. inadai* and the saprophyte, *L. biflexa* also seem to lack *rmlC* and *rmlD* homologs, it is also biologically plausible that *L. licerasiae* truly lacks either gene. A TBLASTN search against the *L. licerasiae* genomes failed to produce any significant alignments, thus it is does not seem that the genes were missed. *L. licerasiae* does possess the enzymes necessary to synthesize GDP-d-rhamnose from GDP-d-mannose, *gmd* (LEP1GSC185_1627; LlicsVM_010100003480) and *rmd* (LEP1GSC185_1109; LlicsVM_010100011485). Although rare, other pathogens such as *Pseudomonas aeruginosa* have been shown to produce LPS containing d-rhamnose [91]; therefore, it is possible that *L. licerasiae* produces d-rhamnose, but this needs to be confirmed experimentally.

The polymerase (*wzy*) and chain length determinant (*wzz*) genes are not observed in the proposed O-antigen locus, but may be located elsewhere in the *L. licerasiae* genome. These genes may be difficult to identify by homology due to their membrane protein nature. There are two identified *wzy* homologs in *L. licerasiae* with candidate orthologs in *L. interrogans* and *L. borgpetersenii*. There are no obvious *wzz* homologs in the *L. licerasiae* genome. The formal possibility exists that this O-antigen consists of only a single repeat, obviating the need for *wzy* and *wzz* genes, but this would be unprecedented if true.

### Evidence for lateral gene transfer in *L. licerasiae*

Seven putative genomic islands in *L. licerasiae* ranging in size from 5 kb to ∼36 kb (Table 4) were identified, the longest of which coincides with the previously mentioned cryptic prophage in sv. Lai and Copenhageni [64]. In addition, we found 28 putative type II toxin-antitoxin systems (TASs) in the VAR010 genome (Table 5). TASs belong to the prokaryotic mobilome as they are extensively, if not preferentially, spread via plasmid-mediated LGT [92]. Like many, if not most of the mobilome members, the TASs are not simply mobile, but appear to behave like selfish elements. If a mobile genetic element encoding a TAS is lost during cell division, the concentrations of the labile antitoxin rapidly decreases, allowing the toxin, which is more stable, to kill the cell. Thus, TASs contribute to the stable maintenance and dissemination of plasmids and genomic islands in bacterial populations despite the associated fitness cost. In *M. tuberculosis*, 37% of these systems are located on genomic islands [93]. In *L. licerasiae*, 36% (10/28) of the putative type II TASs reside on putative genomic islands, and thus, appear to have been acquired by LGT. Of the *L. licerasiae* type II TASs, *chpK*/*chpI* (Table 5) has been confirmed in *L. interrogans* [94] and appears to be unique to infectious species [95]. *L. interrogans* encodes another four TASs [96]. By contrast, *L. biflexa* str. Ames and Paris possess several TASs (22 and 20 TASs, respectively [97]) much like *L. licerasiae*.

**Table 4:** Location of Putative Genomic Islands (GIs) in *L. licerasiae* VAR010.

Contig	Start	End	Length (bp)	# protein coding genes	TAS (# associated)*	Protein ID	
						First	Last
AHOO02000005§	172542	208590	36048	46	No	LEP1GSC185_0932	LEP1GSC185_0976
AHOO02000009	303086	331349	28263	30	Yes (3)	LEP1GSC185_3852	LEP1GSC185_3880
AHOO02000013	449819	471528	21709	23	No	LEP1GSC185_2782	LEP1GSC185_2803
AHOO02000009	620	8558	7938	15	Yes (7)	LEP1GSC185_3556	LEP1GSC185_3570
AHOO02000005	628935	635409	6474	6	No	LEP1GSC185_1388	LEP1GSC185_1392
AHOO02000014	64218	70265	6047	9	No	LEP1GSC185_0603	LEP1GSC185_0612
AHOO02000008	41316	46316	5000	7	No	LEP1GSC185_3455	LEP1GSC185_3460

* Putative type II toxin-antitoxin system.

§ Qin et. Lysogen.

**Table 5:** Putative Type II toxin-antitoxin systems (TAS's) in *L. licerasiae* VAR010.

Locus	Toxin Family	Annotation		Antitoxin Family	Annotation
LEP1GSC185_0180	Aha1	conserved hypothetical protein	LEP1GSC185_0181	ArsR	toxin-antitoxin system, antitoxin component, ArsR family
LEP1GSC185_0262	Aha1	conserved hypothetical protein	LEP1GSC185_0263	ArsR	transcriptional regulator, ArsR family
LEP1GSC185_0307	PIN	PIN domain protein	LEP1GSC185_0308	Phd/YefM	prevent-host-death family protein
LEP1GSC185_0418	PIN	PIN domain protein	LEP1GSC185_0416	Phd/YefM	prevent-host-death family protein
LEP1GSC185_0630	PIN	toxin-antitoxin system, toxin component, PIN family	LEP1GSC185_0631	Phd/YefM	antitoxin Phd/YefM, type II toxin-antitoxin system
LEP1GSC185_1922	PIN	PIN domain protein	LEP1GSC185_1923	DUF2191	PF09957 family protein
LEP1GSC185_2251	PIN	PIN domain protein	LEP1GSC185_2250	Phd/YefM	conserved hypothetical protein
LEP1GSC185_2325	Aha1	conserved hypothetical protein	LEP1GSC185_2324	HTH	DNA-binding helix-turn-helix protein
LEP1GSC185_2580	PIN	PIN domain protein	LEP1GSC185_2582	RHH	toxin-antitoxin system, antitoxin component, ribbon-helix-helix domain protein
LEP1GSC185_2709	Aha1	conserved hypothetical protein	LEP1GSC185_2710	ArsR	MarR family protein
LEP1GSC185_3193	PIN	putative toxin-antitoxin system toxin component, PIN family	LEP1GSC185_3194	RHH	putative toxin-antitoxin system, antitoxin component, ribbon-helix-helix domain protein
LEP1GSC185_3380	Aha1	conserved hypothetical protein	LEP1GSC185_3379	ArsR	DNA-binding helix-turn-helix protein
LEP1GSC185_3451	COG2856	PF06114 domain protein	LEP1GSC185_3450	XRE	conserved hypothetical protein
LEP1GSC185_3530	PIN	toxin-antitoxin system toxin component, PIN family	LEP1GSC185_3529	RHH	ribbon-helix-helix protein, CopG family
LEP1GSC185_3543	ChpK	PemK-like protein	LEP1GSC185_3542	ChpI	ribbon-helix-helix protein, CopG family
LEP1GSC185_3550	PIN	PIN domain protein	LEP1GSC185_3549	MazE	antidote-toxin recognition MazE
LEP1GSC185_3553	PIN	toxin-antitoxin system, toxin component, PIN family	LEP1GSC185_3552	COG2442	putative toxin-antitoxin system, antitoxin component
LEP1GSC185_3555	MazF	putative toxin-antitoxin system, toxin component, MazF family	LEP1GSC185_3554	RHH	ribbon-helix-helix protein, CopG family
LEP1GSC185_3557	PIN	PIN domain protein	LEP1GSC185_3556	RHH	toxin-antitoxin system, antitoxin component, ribbon-helix-helix domain protein
LEP1GSC185_3559	PIN	toxin-antitoxin system toxin component, PIN family	LEP1GSC185_3558	RHH	ribbon-helix-helix protein, CopG family
LEP1GSC185_3561	PIN	PIN domain protein	LEP1GSC185_3560	AbrB	transcriptional regulator, AbrB family
LEP1GSC185_3562	UNK	putative toxin-antitoxin system, toxin component	LEP1GSC185_3563	RHH	toxin-antitoxin system, antitoxin component, ribbon-helix-helix domain protein
LEP1GSC185_3564	COG2929	PF04365 family protein	LEP1GSC185_3565	RHH	toxin-antitoxin system, antitoxin component, ribbon-helix-helix domain protein
LEP1GSC185_3566	PIN	PIN domain protein	NA*	UNK	hypothetical protein
LEP1GSC185_3584	Aha1	conserved hypothetical protein	LEP1GSC185_3585	ArsR	MarR family protein
NA	DUF497	hypothetical protein LlicsVM_07915	LEP1GSC185_3866	RHH	toxin-antitoxin system, antitoxin component, ribbon-helix-helix domain protein
NA	HigB	plasmid maintenance killer protein	LEP1GSC185_3875	HigA	addiction module antidote protein HigA (higA)
LEP1GSC185_3880	PIN	toxin-antitoxin system toxin component, PIN family	LEP1GSC185_3879	RHH	ribbon-helix-helix protein, CopG family

* Manually annotated—missed by automated gene caller.

As additional independent evidence of lateral transfer, more than half of the *L. licerasiae*-specific CDS have no or poor homology with other leptospiral proteins. These include phosphate, chromium and molybdate transport systems. Of these proteins, most have homology with non-invasive environmental bacteria including *Sorangium cellulosum* [6 proteins], *Bdellovibrio bacteriovirus* [6 proteins] and *Haliscomenobacter hydrossis* [5 proteins]. While IS elements appear to be major contributors to genomic diversification in pathogenic *Leptospira*, which may possess more than 20 insertion sequence (IS) elements [19], the relative lack of IS elements in the *L. licerasiae* and *L. biflexa* genomes would suggest that genomic diversity where it exists is a result of different mechanisms. The phylogenetic origins of the laterally transferred genes, suggest that *L. licerasiae* is able to exchange genetic material with non-invasive environmental bacteria, whether this species can become naturally competent remains to be determined.

### Conclusion

This study bridges a major gap in our knowledge of leptospiral biology and addresses a key question in the field regarding the pathogenic potential of the intermediate clade of *Leptospira* [9]. The data presented here 1) demonstrate that *L. licerasiae* is more closely related to pathogenic than to saprophytic *Leptospira*; 2) provide insight into the genomic bases for its infectiousness and unique antigenic characteristics; and 3) support the denomination of the intermediate clade as ‘intermediately pathogenic’ and its consideration as a transitional group between saprophytes and pathogens.

Future comparative genomic analysis of the complete set of *Leptospira* species will provide deeper large-scale insights into the evolution, biology and evolution of virulence of this genus of spirochetes, and guide new experimental directions.

## Supporting Information

Table S1 **Identification of 1211 conserved** ***Leptospira licerasiae*** **proteins with no orthologs in** ***L. interrogans*** **,** ***L. borgpetersenii*** **or** ***L. biflexa*** **.**
(XLSX)

Table S2 **Identification of 452 conserved pathogen-specific proteins with 315 assigned a putative function by domain homology searches.**
(XLSX)
